# Coexistence of Intracranial and Spinal Cord Cavernous Malformations Predict Aggressive Clinical Presentation

**DOI:** 10.3389/fneur.2019.00618

**Published:** 2019-06-13

**Authors:** Jian Ren, Tao Hong, Chuan He, Liyong Sun, Xiaoyu Li, Yongjie Ma, Jiaxing Yu, Feng Ling, Hongqi Zhang

**Affiliations:** Department of Neurosurgery, China International Neuroscience Institute, Xuanwu Hospital, Capital Medical University, Beijing, China

**Keywords:** cavernous malformations, hemorrhage, intracranial, natural history, spinal cord, stroke

## Abstract

**Background:** Patients with spinal cord cavernous malformations (CMs) are at increased risk for multiple neuraxis CMs. Few studies focused on the natural history of patients with coexistence of intracranial and spinal cord CMs.

**Methods:** Forty patients who underwent both intracranial and spinal MR imaging from a single center were reviewed retrospectively. American Spinal Injury Association (ASIA) impairment scale was used to evaluate neurological and disability status.

**Results:** The median age of the 40 patients was 40.6 years old (range 9–69), and the male-to-female ratio was 2.1:1. The mean size of the intramedullary lesions was 10.1 ± 5.8 mm (range, 3.0–34.0 mm). Six patients (15%) harbored at least one intracranial lesion. Five of the 6 patients (83.3%) suffered aggressive clinical presentations with severe neurological and disability status; in patients with sporadic spinal CMs, the proportion was 26.5% (9 of 34). Coexistence of intracranial and spinal cord CMs is more likely to follow an aggressive course (*P* = 0.031, OR = 19.547, 95% CI = 1.322–289.123). In the postoperative long-term follow up, the unfavorable rate of patients with associated intracranial CMs was significantly higher than that of patients with sporadic spinal cord CMs (*P* = 0.049).

**Conclusions:** The prevalence of associated intracranial CMs in patients with spinal cord CMs was 15%. Coexistence of intracranial and spinal cord CMs is more likely to follow an aggressive course. This study highlights the necessity of intracranial MR imaging for patients with spinal cord CMs to benefit to the predicting prognosis and selection of treatment strategies.

## Introduction

Cavernous malformations (CMs) are low-flow vascular malformations that are the second most common type of central nervous system vascular lesion (1). CMs have an incidence of approximately 0.4–0.8% in the general population (1). These lesions mostly occur in an intracranial location. Spinal cord CMs are rare and constitute 5–12% of all spinal vascular lesions (2). CMs in spinal cord are more aggressive because of the narrow spinal cavity, which has a low tolerance for space-occupying lesions (3–5).

CMs tend to fall into two categories: familial (inherited) forms and sporadic forms. It was reported in the literature that of the patients with CMs, 10–15% had a familial (inherited) form (2). Patients with spinal cord CMs are at increased risk for multiple neuraxis CMs (6–8). In contrast to the intracranial CMs, the natural history of spinal cord CMs has been less thoroughly established for the rarity (1, 2, 9–11). Few studies focused on the natural history of patients with coexistence of intracranial and spinal cord CMs. No previous literature found any difference in natural history or treatment outcomes between sporadic spinal cord CMs and coexistence of intracranial and spinal cord CMs, which was mainly due to the rare occurrence of spinal cord CMs (4, 7).

Our initial experiences of spinal cord CMs were reported previously (12–14). In this study, we provide a more detailed analysis of the prevalence, natural history and postoperative long-term outcomes of patients with coexistence of intracranial CMs and spinal cord CMs.

## Materials and Methods

### Patients

Between January 2002 and September 2017, 40 patients with spinal cord CMs who underwent both intracranial and spinal MR imaging were reviewed retrospectively extracted from a consecutive series of 254 patients with spinal cord CMs seen in a single institution. The local ethics board of Xuanwu Hospital, Capital Medical University approved this study, which was performed in accordance with the ethical standards of the 1964 Declaration of Helsinki. The patient consent was informed and written for all patients enrolled in this study. Considering the low tolerance for space-occupying lesions and a lifelong hemorrhage risk, the treatment strategies for ISCCMs are more proactive. In our institution, all symptomatic spinal cord CMs patients even those with transient or minimally symptomatic status, undergo surgical resection as the optimal treatment (14).

### Definition of Variables

We used the American Spinal Injury Association (ASIA) impairment scale was to evaluate neurological and disability status. Aggressive clinical presentation which was presented as severe neurological and disability status was defined as a grade on the ASIA scale from A to C. We defined long-term follow-up as a last follow-up time at least 6 months after surgery for surgical group or discharge for conservative group. We defined an unfavorable outcome at follow-up as ASIA grade of A to C.

Criteria for overt hemorrhage of CMs have been proposed (15). We strictly defined a hemorrhage event as a symptomatic event with radiographic evidence of overt hemorrhage.

### Statistics

We used Fisher's exact test or Pearson's χ^2^-test (with or without Yates continuity correction) for categorical variables and Student's *t*-test for continuous variables to evaluate differences in clinical variables and outcomes. We calculated the annual stratified retrospective hemorrhage rate based on the frequencies of hemorrhage events and neurological events and patient age in years. The rehemorrhage rate was calculated based on the frequency of rehemorrhage events and follow-up in years. Logistic regression analysis was used to assess the impact of multiple variables on binary clinical presentation (aggressive vs. mild) and factors predicting aggressive clinical presentation. All analyses were performed under the guidance of an epidemiologist using SPSS software (version 25, IBM Corp., Armonk, New York, USA). All *P*-values were 2-sided, and we considered statistical significance as a value of *P* < 0.05.

## Results

Of the 40 patient with spinal cord CMs who underwent intracranial MR imaging, the median age on admission was 40.6 years old (range 9–69), and the male-to-female ratio was 2.1:1 (27 males and 13 females). The overall mean size of the symptomatic lesions at presentation was 10.1 ± 5.8 mm (range, 3.0–34.0 mm).

All the 40 patients harbored one lesion in the spinal cord. The spinal level was cervical in 17 patients (42.5%) and thoracic in 23 patients (57.5%). Six (15.0%) of the 40 patients were found harboring at least one intracranial lesion. The prevalence of associated intracranial CMs in patients with spinal cord CMs was 15%. All the 6 patients with associated intracranial CMs were performed surgical resection of the spinal cord lesions. All the intracranial CMs were managed conservatively. The demographic or CM- related characteristics of the 6 patients were summarized in Table 1.

**Table 1:** Characteristics of 6 patients with coexistence of intracranial and spinal cord CMs^a^.

**Case No**.	**Location of spinal cord CM**	**Location of intracranial CM**	**Symptoms**	**ASIA grade**
1	T5–T6	Multiple in cerebrum and cerebellum	Complete paralysis	A
2	T2	Lt temporal, Lt corona radiata	Complete paralysis	A
3	T11–T12	Multiple in cerebrum and cerebellum	Complete paralysis	A
4	T6	Lt caput nuclei caudati	Bi LE weakness and hypesthesia, Bd	B
5	T5	Cerebellum and brain stem	Bi LE weakness and hypesthesia	D
6	T11–T12	Rt parietal	Bi LE weakness and hypesthesia, Bd	C

a *No, number; Bi, bilateral; Lt, left; Rt, right; LE, lower extremity; Bd, Bowel/bladder dysfunction*.

Among the 34 patients with sporadic spinal cord CMs, 26 patients underwent microsurgical resections, and 8 patients underwent conservative management.

### Presentation

Typically, the onset of multiple neurological signs or symptoms was abrupt or progressive caused by acute overt hemorrhage or repetitive intralesional microhemorrhages. On admission, 36 (90.0%) patients had a history of acute onset of various type of neurological decline, including rapidly declining, progressive worsening, transient or minimally symptomatic status, and once or discretely occurring episodes. Four (10.0%) patients presented with slowly progressive neurological decline with no acute onset of signs or symptoms. The most common presenting signs and symptoms were sensory deficits (92.5%), weakness (85.0%), bowel/bladder dysfunction (42.5%), and pain (40.0%). On admission, disability was grade A in 4 (10.0%) of the patients, grade B in 3 (7.5%), grade C in 7 (17.5%), grade D in 25 (62.5%), and grade E in 1 (2.5%) according to the ASIA scale. Eighty-three (32.7%) of these patients presented with a severe neurological and disability status.

Baseline data of the 6 patients with associated intracranial CMs was compared with 34 patients with sporadic spinal cord CMs. The two groups did not differ on demographic or CM- related characteristic assessed except for the location of the spinal lesions (*P* = 0.030) and neurological and disability status (*P* = 0.026). Of the 6 patients with associated intracranial CMs, 5 patients (83.3%) suffered aggressive clinical presentation with severe neurological and disability status (ASIA grade from A to C). While of the 34 patients with sporadic spinal cord CMs, only 9 patients (26.5%) suffered aggressive clinical presentation. Associated intracranial CMs was more likely to follow an aggressive course (*P* = 0.026).

### Hemorrhage Rates

We identified 39 overt hemorrhage events among 1,624 patient-years of life, and we retrospectively calculated the annual overt hemorrhage rate of symptomatic as 2.4%/patient/year. After initial overt hemorrhage events, we identified 4 re-hemorrhage events across 108.2 patient-years of life; thus, the annual overt re-hemorrhage rate was 3.7%/patient/year, which was higher than the initial hemorrhage rate (3.0%/patient/year).

### Factors Predicting Aggressive Clinical Presentation

Parameters before treatment were analyzed separately using univariate and multivariate analyses to determine predictors of aggressive clinical presentation. A univariate analysis revealed that coexistence of intracranial and spinal cord CMs was associated with an aggressive clinical presentation, with coexistence of intracranial and spinal cord CMs associated with a statistically significant increase in the likelihood of a severe neurological and disability status (*P* = 0.024, OR = 13.889, 95% CI = 1.423–135.544). In the multivariate analysis, coexistence of intracranial and spinal cord CMs was also a significant predictor of an aggressive clinical presentation (*P* = 0.031, OR = 19.547, 95% CI = 1.322–289.123; Table 2).

**Table 2:** Factors predicting aggressive clinical presentation of 40 patients with spinal cord CMs.

**Variable**	**OR**	**95% Cl**	***P*-value**
**UNIVARIATE ANALYSIS**			
Female	3.3	0.8–13.4	0.089
Age, per each additional year	1.0	0.9–1.0	0.356
Thoracic and lumber located	2.5	0.6–10.0	0.197
Size, per 0.1 cm increase	1.6	0.5–4.9	0.453
Coexistence of intracranial and spinal CMs	13.9	1.4–135.5	0.024
**MULTIVARIATE ANALYSIS**			
Female	4.7	0.9–25.3	0.074
Age, per each additional year	1.0	0.9–1.0	0.196
Thoracic and lumber located	1.4	0.3–8.3	0.680
Size, per 0.1 cm increase	1.6	0.4–5.5	0.490
Coexistence of intracranial and spinal CMs	19.5	1.3–289.1	0.031

We found no evidence that age, gender, size or location of lesions had any significant difference in the likelihood of a severe neurological and disability status (Table 2).

### Postoperative Long-Term Outcomes

Long-term follow up was obtained in 37 patients (92.5%), including 6 with associated intracranial CMs and 31 with sporadic spinal cord CMs (7 conservatively managed, and 24 surgical treated). The mean follow-up duration of the surgical group (*n* = 24) was 40.5 ± 38.9 months. The mean follow-up duration of the conservative group (*n* = 7) was 72.0 ± 25.9 months.

Compared with preoperative neurological status, the postoperative follow-up outcomes in 6 patients with associated intracranial CMs demonstrated that 5 patients (60.0%) were stable, 1 (33.3%) improved and 0 (0.0%) worsened; in 24 patients with sporadic spinal cord CMs, the proportions were 70.8, 25.0, and 4.2%, respectively. Four (66.7%) in 6 patients with associated intracranial CMs and 5 (20.8%) in 24 patients with sporadic spinal cord CMs had unfavorable outcomes. The unfavorable rate of patients with associated intracranial CMs was significantly higher than that of patients with sporadic spinal cord CMs (*P* = 0.049, Fisher exact test).

Of the 7 conservatively managed patients, 4 patients (57.1%) were stable, 1 (14.3%) improved, and 2 (28.5%) worsened. The surgical group and conservative group did not differ in prognosis of long-term follow-up outcomes (*P* = 0.156) and favorable outcomes (*P* = 0.711).

## Discussion

Spinal cord CMs are more aggressive for the narrow spinal cavity contributing to a low tolerance for space-occupying lesions (4). Patients with spinal cord CMs are a heterogeneous group, exhibiting a wide range of clinical presentation and totally different outcomes, and making it difficult to predict further hemorrhagic and neurological risk after the initial onset in CMs (9).

Previous studies have found that patients with spinal cord CMs are at increased risk for multiple neuraxis CMs (6–8). In the literature, approximately 16.5 to 42% of patients with spinal cord CMs also have intracranial lesions, and these rates are slightly higher than the rate found in our cohort (2, 4, 7).

To the best of our knowledge, no previous literatures found any difference in natural history or treatment outcomes between sporadic spinal cord CMs and coexistence of intracranial and spinal cord CMs, which was mainly due to the rare occurrence of spinal cord CMs (4, 7). In the present study, we first showed that coexistence of intracranial CMs and spinal cord CMs predict a statistically significant increase in the likelihood of aggressive clinical presentation and unfavorable outcomes than sporadic spinal cord CMs. The coexistence of spinal cord CMs and intracranial CMs was typically occur in patients with the familial form of CMs (7). Up to 80–90% of familial CMs are multiple lesions. Multiplicity of intracranial CMs were found increasing the risk of hemorrhage and the familial form of CMs is more aggressive (16–19). We speculate that the aggressive characteristic of coexistence of intracranial and spinal cord CMs is related to its familial form of CMs, which was found more aggressive than sporadic CM in previous literature (18, 19).

These findings help draw a clearer distinction between the wide range of clinical presentation and totally different outcomes in the patients with spinal cord CMs and highlight the necessity of intracranial MR imaging for patients with spinal cord CMs to benefit to the predicting prognosis and selection of treatment strategies.

There are limitations to this study that should be mentioned to allow an accurate interpretation of our findings. A selection bias that patients with aggressive spinal cord CMs were more likely to receive intracranial MR imaging may have resulted in patients with spinal cord CMs with a serious clinical presentation being overrepresented in the present study. In our study, the findings that coexistence of intracranial and spinal cord CMs were more aggressive was based on a comparison with the other 34 patients with sporadic CMs who also received intracranial MR imaging and the bias between the groups was minimized. Another limitation of the study is its relative small sample size and lack of a prospective design because of the rare occurrence of spinal cord CMs. To avoid these limitations, the investigation with prospective design and more patients is necessary in the future.

## Conclusions

The prevalence of associated intracranial CMs in patients with spinal cord CMs was 15%. Coexistence of intracranial and spinal cord CMs is more likely to follow an aggressive course. This study highlights the necessity of intracranial MR imaging for patients with spinal cord CMs to benefit to the predicting prognosis and selection of treatment strategies.

## Ethics Statement

The local ethics board of Xuanwu Hospital, Capital Medical University approved this study, which was performed in accordance with the ethical standards of the 1964 Declaration of Helsinki. The patient consent was informed and written for all patients enrolled in this study.

## Author Contributions

JR, TH, CH, LS, XL, YM, JY, FL, and HZ have substantially contributed to the conception, design, analysis, and interpretation of the data as well as to drafting the article and revising it critically. All authors have read and approved the final version of the manuscript.

### Conflict of Interest Statement

The authors declare that the research was conducted in the absence of any commercial or financial relationships that could be construed as a potential conflict of interest.
